# Opposing presynaptic roles of BDNF and ProBDNF in the regulation of persistent activity in the entorhinal cortex

**DOI:** 10.1186/s13041-016-0203-9

**Published:** 2016-03-01

**Authors:** Julien Gibon, Philip A. Barker, Philippe Séguéla

**Affiliations:** Department of Neurology and Neurosurgery, Montreal Neurological Institute, McGill University, Suite 778, Montreal, Quebec H3A 2B4 Canada

**Keywords:** Persistent firing, Neurotrophin, Working memory, Spatial memory, Acetylcholine, mGluR5, p75NTR, TrkB

## Abstract

**Background:**

Sustained, persistent firing (PF) of cortical pyramidal neurons following a short depolarization is a crucial cellular mechanism required for spatial and working memory. Pyramidal neurons in the superficial and deep layers of the medial and lateral entorhinal cortex (EC) display this property of prolonged firing activity. Here, we focused on the regulation of this activity in EC neurons by mature brain derived neurotrophic factor (BDNF) and its precursor proBDNF.

**Results:**

Using patch clamp electrophysiology in acute mouse cortical slices, we observed that BDNF facilitates cholinergic PF in pyramidal neurons in layer V of the medial EC. Inhibition of TrkB with K252a blocks the potentiating effect of BDNF whereas inhibition of p75NTR with function-blocking antibodies does not. By recording spontaneous excitatory post-synaptic currents (sEPSC), we find that BDNF acts pre-synaptically via TrkB to increase glutamate release whereas proBDNF acting via p75NTR acts to reduce it. MPEP abolished the facilitating effect of BDNF on PF, demonstrating that the metabotropic glutamate receptor mGluR5 plays a critical role in the BDNF effect. In contrast, paired pulse ratio and EPSC measurements indicated that proBDNF, via presynaptic p75NTR, is a negative regulator of glutamate release in the EC.

**Conclusions:**

Taken together, our findings demonstrate that the BDNF/TrkB pathway facilitates persistent activity whereas the proBDNF/p75NTR pathway inhibits this mnemonic property of entorhinal pyramidal neurons.

## Background

Neuronal persistent activity or persistent firing (PF) is observed in several brain areas [1]. In the temporal lobe, pyramidal neurons from the entorhinal cortex [2–5], from hippocampal CA1 [6, 7], CA3 [8] and subiculum [9, 10] are able to sustain their firing activity after a brief stimulus. During working memory tasks, principal neurons in the entorhinal cortex (EC) of rats and primates display persistent firing (PF) and this activity represent a cellular feature with intrinsic and network properties that are essential for short-term or working memory [11, 12]. In the medial EC, persistent activity is proposed to underlie the process of spatial mapping generated by the grid cells [13, 14]. The EC receives cholinergic inputs from the medial septum [15], and cholinergic modulation via muscarinic receptors is essential for working memory and PF [16]. Muscarinic receptor-induced changes in the intrinsic firing properties of cortical neurons via recruitment of calcium-permeable TRP channel and represents an important form of neuromodulation [2–4]. PF has been shown to be involved in key mnemonic processes but whether this activity is regulated by other endogenous neuromodulators remains unknown. In this study, we investigated the role of the neurotrophins brain-derived neurotrophic factor (BDNF) and its precursor proBDNF in the control of PF.

BDNF has been shown to potentiate hippocampal long term potentiation (LTP), to increase the release of glutamate [17] and to play an important role in formation and recall of spatial memory [18]. BDNF belongs to the family of neurotrophins which is composed, in mammals, of nerve growth factor (NGF), neurotrophin 3 (NT3) and neurotrophin 4/5 (NT4/5) [19]. BDNF is initially synthesized as a precursor, proBDNF, which is proteolytically cleaved to generate mature BDNF [20]. Two receptors are known to bind BDNF: TrkB and p75 neurotrophin receptor (p75NTR). TrkB belongs to the receptor tyrosine kinase family and can also bind NT4; p75NTR can bind all the neurotrophins and can acts as a co-receptor for Trk receptors [21, 22]. Proneurotrophins bind to receptor complexes composed of p75NTR and VPS10 family members. BDNF-containing secretory vesicles are present in axon terminals but their presence in dendrites of glutamatergic neurons is still debated [23–25]. TrkB is expressed on pre- and post-synaptic compartments of central glutamatergic synapses and BDNF has emerged as a major regulator of synaptic plasticity [26].

Here, we investigated the role of BDNF and proBDNF on the persistent firing properties of pyramidal neurons in the layer V of EC. ProBDNF and BDNF have been described as yin-yang molecules with opposing effects on cell function [19]. Our working hypothesis was that BDNF and proBDNF exert differential neuromodulatory effects through preferred activation of the TrkB and p75NTR transduction pathway, respectively. Based on patch clamp electrophysiology and pharmacology in acute mouse brain slices, our findings indicate that mature BDNF and proBDNF play opposite roles in the regulation of PF in cortical pyramidal neurons.

## Results

**Fig. 1:**
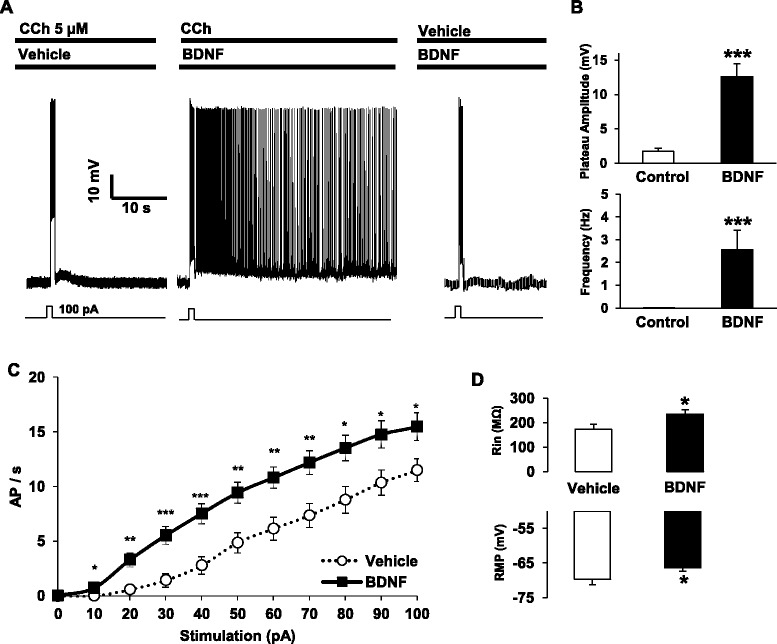
BDNF facilitates persistent firing and increases excitability of EC layer V pyramidal neurons. **a** BDNF (50 ng/ml, *n* = 6) or sub-threshold carbachol (CCh, *n* = 16) do not induce persistent firing in EC pyramidal neurons following a brief stimulus (100 pA, 1 s) whereas 90 min incubation with BDNF (50 ng/ml) facilitates persistent activity in presence of CCh (*n* = 11). **b** Quantification of plateau potential amplitude (*** *p* = 0.0002, Mann-Whitney test) and frequency (*** *p* < 0.0001, Mann-Whitney test) of persistent firing. **c** Input/output curve from pyramidal neurons in slices pre-incubated with vehicle (*n* = 14) or with BDNF 90 min (*n* = 21), * *p* < 0.05, ** *p* < 0.01, *** *p* < 0.001, T-test. **d** Input resistance and resting membrane potential from the pyramidal neurons recorded in C (Rin: * *p* = 0.028; RMP: * *p* = 0.0411, Mann-Whitney test)

We recently showed that the proBDNF/p75NTR down regulates excitability and firing of layer V pyramidal neurons in the EC [27]. In the present study, we tested the hypothesis that BDNF would facilitate the cholinergic persistent activity of layer V principal neurons. Cortical slices were incubated with BDNF (50 ng/ml) then subjected to a brief stimulus (100 pA, 1 s). Figure 1a shows that exposure to BDNF alone or CCh alone failed to induce persistent firing following a depolarization pulse whereas pre-incubation with BDNF in presence of CCh 5 μM allowed neurons to reach the persistent firing state. BDNF significantly increased the amplitude of the plateau potential (CCh alone: 1.71 +/− 0.48 mV; + BDNF: 12.71 +/− 1.88 mV) and significantly increased the frequency of the persistent activity (CCh alone: 0.007 +/− 0.006 Hz; + BDNF: 2.80 +/− 0.89 Hz) (Fig. 1b). To determine if the effect of BDNF was due to an increase of neuronal excitability, as previously shown [28, 29], we recorded input/output curves from pyramidal neurons pre-treated with vehicle or BDNF. BDNF clearly increased the number of action potentials for each stimulus tested between 10 and 100 pA (Fig. 1c). BDNF treatment also increased the input resistance and depolarized the resting membrane potential of pyramidal neurons in EC layer V (Fig. 1d). Together, these data indicate that BDNF increases cell excitability and potentiates persistent firing in the EC.

**Fig. 2:**
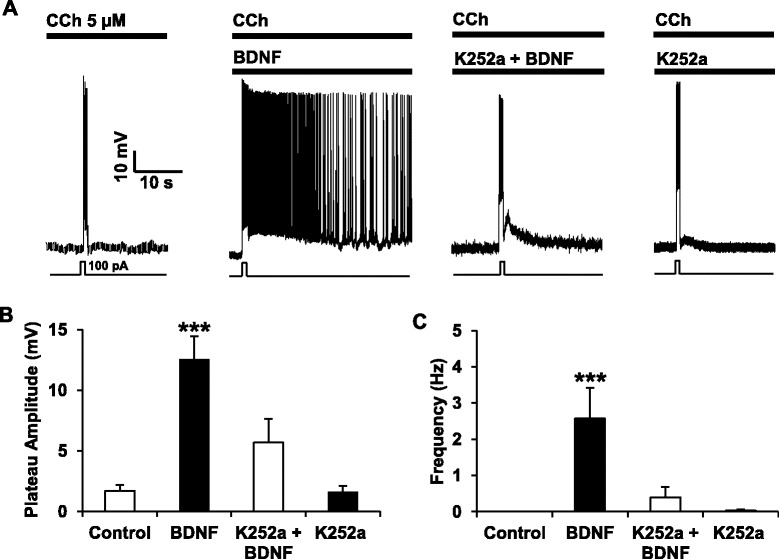
TrkB mediates the BDNF-induced facilitation of persistent firing. **a** Incubation with K252a (200 nM) 30 min before the BDNF treatment fully blocks the induction of persistent firing whereas K252a alone has no effect on persistent activity. Quantification of plateau potential amplitudes (**b**) and firing frequencies (**c**) of persistent firing, *** *p* < 0.001, ANOVA followed by Bonferroni multiple comparison

**Fig. 3:**
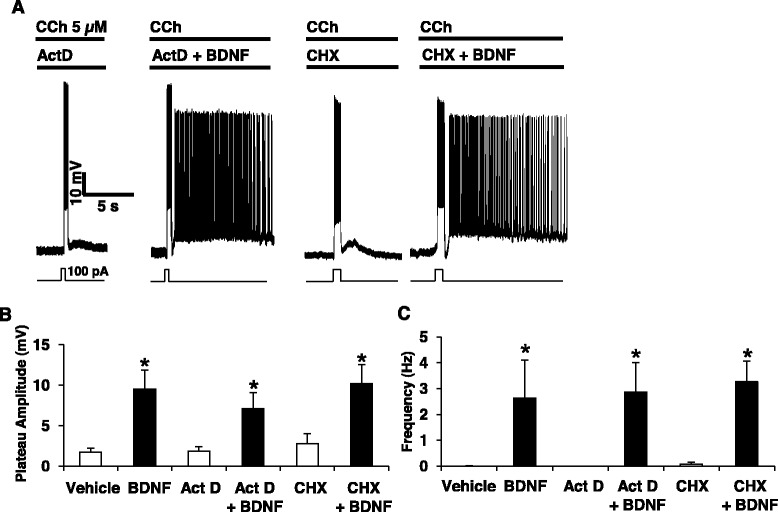
Facilitatory effects of BDNF on persistent firing do not require *de novo* mRNA or protein synthesis. **a** Pre-incubation of slices with actinomycin D (ActD, 25 μM, 60 min) or cycloheximide (CHX, 1 μg/ml, 60 min) does not prevent BDNF facilitation of persistent firing induced by 5 μM CCh. Quantification of plateau potential amplitudes (**b**) and firing frequencies (**c**), * *p* < 0.05, ANOVA followed by Bonferroni multiple comparison

**Fig. 4:**
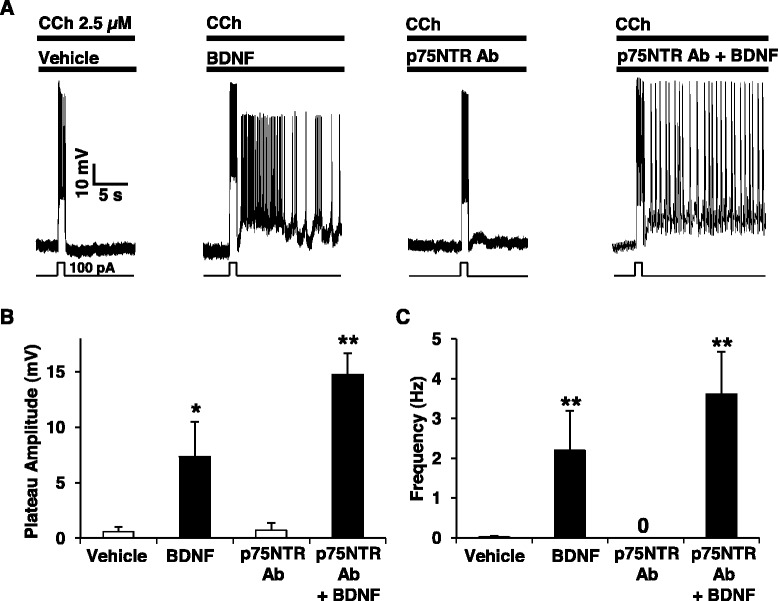
BDNF-induced facilitation of persistent firing is independent of p75NTR. **a** Pre-incubation with p75NTR function-blocking antibodies does not occlude BDNF potentiating effects on persistent firing elicited at 2.5 μM CCh. Quantification of plateau potential amplitudes (**b**) and firing frequencies (**c**). * *p* < 0.05, *** *p* < 0.001 ANOVA followed by Tukey multiple comparison. Statistics are shown for vehicle (*n* = 4) vs. BDNF (*n* = 4) and p75NTR Ab (*n* = 3) vs. p75NTR Ab + BDNF (*n* = 3)

BDNF can bind to TrkB and to p75NTR [19] and we next established the transduction pathway required for facilitation of PF by BDNF. Using the receptor tyrosine kinase antagonist K252a at a low concentration (200 nM), we were able to completely block the effect of BDNF on PF (Fig. 2a). Interestingly, we still observed a large transient after-depolarization but we did not observe PF when slices are treated with K252a prior to the BDNF treatment (Fig. 2a–c). BDNF facilitates PF after several minutes of incubation and to check if the facilitation of PF by BDNF requires mRNA synthesis and/or protein synthesis, we incubated the cortical slices with the transcription inhibitor actinomycin D (ActD) or the translation inhibitor cycloheximide (CHX) prior to BDNF. We observed that ActD or CHX did not block the potentiating effect of BDNF on PF (Fig. 3a–c) indicating that neither *de novo* transcripts nor *de novo* proteins are required for BDNF-induced facilitation of PF. We then investigated the contribution of p75NTR to the BDNF effects on PF. As deletion or blockade of p75NTR facilitates the expression of cholinergic PF [27], we decreased the concentration of CCh to a point (2.5 μM) where the blockade of p75NTR with function-blocking antibodies does not induce PF. At this concentration of CCh, blockade of p75NTR has no effect while BDNF is still able to facilitate PF (Fig. 4a), making this an optimal condition to test if p75NTR activation has any impact on the facilitation of PF by BDNF (Fig. 4a). Pre-treatment of the slices with antibodies targeting the extracellular domain of p75NTR did not block BDNF-dependent PF (Fig. 2a). On the contrary, firing was more stable and both firing frequency and plateau potential amplitude were increased when p75NTR was blocked (BDNF alone: 2.2 +/−0.98 Hz, 7.38 +/− 3.1 mV; BDNF + p75NTR Ab: 3.61 +/− 1.06 Hz, 14.77 +/− 1.89 mV) (Fig. 4b, c), consistent with the notion that p75NTR does have an inhibitory role on neuronal firing [27, 30].

**Fig. 5:**
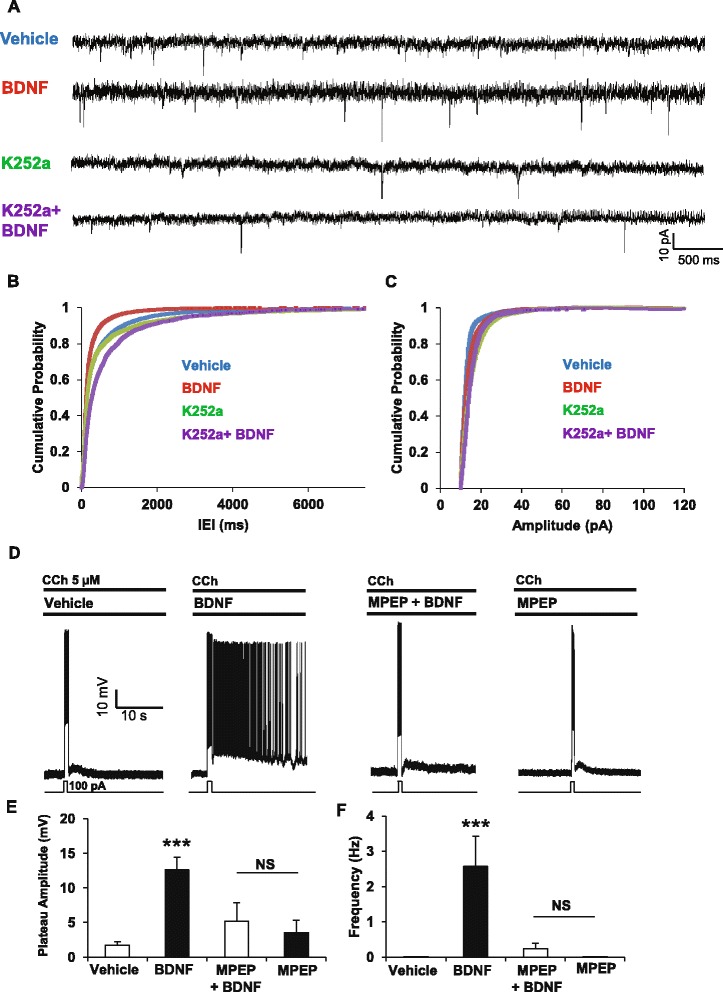
BDNF increases glutamate release and mGluR5 is necessary for BDNF-induced facilitation of cholinergic persistent firing. **a** Representative traces of voltage-clamp recordings of sEPSC from slices treated with vehicle (*n* = 13), BDNF (50 ng/ml, *n* = 14), K252a (200 nM, *n* = 9) and K252a + BDNF (*n* = 6). **b** Cumulative probability plot of sEPSC inter-event-intervals (IEI). **c** Cumulative probability plot of sEPSC amplitudes. **d** MPEP blocks the facilitation of PF normally induced by BDNF in presence of 5 μM CCh. Quantification of plateau potential amplitudes (**e**) and persistent firing frequencies (**f**) (MPEP + BDNF *n* = 5, MPEP alone *n* = 9), *** *p* < 0.001 ANOVA followed by Bonferroni multiple comparison

Persistent firing can result from the activation of postsynaptic metabotropic glutamate receptors [31] and we next examined the effect of BDNF on glutamate release by measuring spontaneous excitatory postsynaptic current (sEPSC) in EC layer V pyramidal neurons (Fig. 5a). Pre-treatment of slices with BDNF (50 ng/ml) decreased inter-event intervals (IEI) without affecting the amplitude of sEPSCs (Fig. 5b, c) and application of the Trk inhibitor K252a at low concentration (200 nM) completely abolished the effect of BDNF on IEI (Fig. 5a). Thus, BDNF acts pre-synaptically via TrkB to increase glutamate release.

Activation of mGluR5 can promote cholinergic PF and induce PF by itself [31] and we therefore investigated the role of mGluR5 in the BDNF-induced facilitation of cholinergic PF. Treatment with the selective mGluR5 antagonist MPEP suppressed the facilitation of cholinergic PF by BDNF (Fig. 5d) and the MPEP pretreatment prevented any significant BDNF-induced increase in plateau potential amplitude (Fig. 5e) or firing frequency (Fig. 5f). Thus, we conclude from these findings that BDNF, via presynaptic TrkB, increases the release of glutamate which in turn acts on postsynaptic Gq-coupled mGluR5 receptors to facilitate the firing of pyramidal neurons in the EC.

**Fig. 6:**
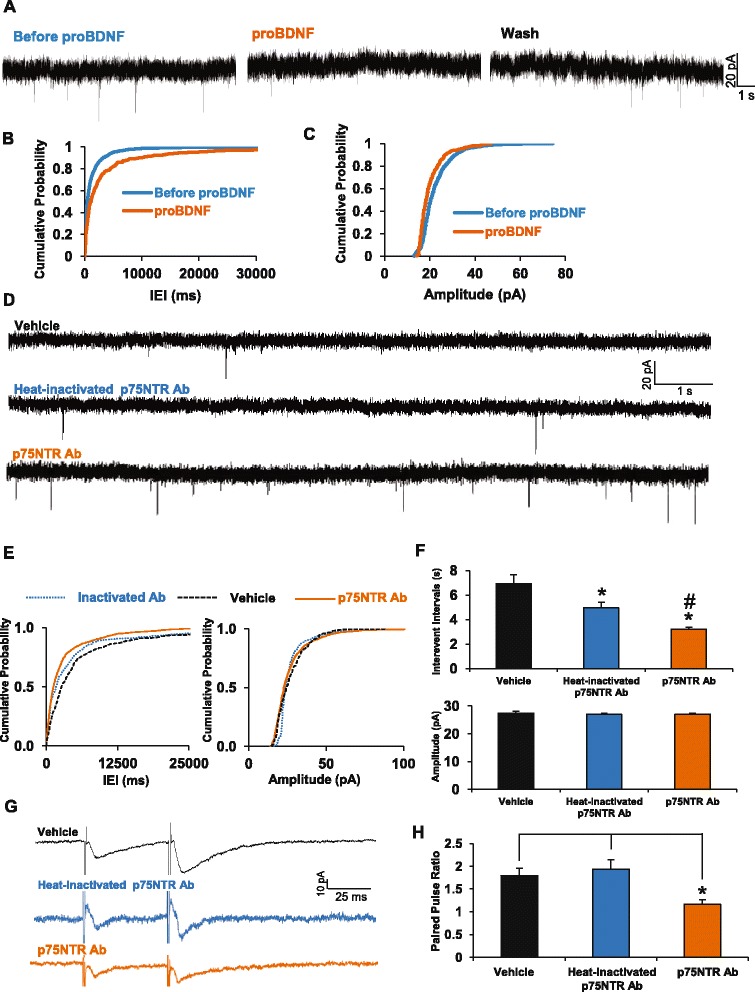
The proBDNF/p75NTR axis controls glutamate release in the entorhinal cortex. **a** Representative traces of voltage-clamp recordings of sEPSCs during 5 min (before proBDNF), followed by 10 min of perfusion with proBDNF (2 ng/ml) and then recorded 5 min (proBDNF), followed by 10 min washout with normal ACSF following by 5 min of recording (Wash) (*n* = 6). **b**, **c** Cumulative plots of inter-event intervals and sEPSC amplitudes. **d** Representative traces of voltage-clamp recording of sEPSCs from slices treated with inactivated (*n* = 11) or active p75NTR function-blocking antibodies (1/500, *n* = 10). **e** Cumulative plots of interevent intervals and sEPSC amplitudes. **f** Means +/- S.E.M. * *p* < 0.05 using Kruskal-Wallis ANOVA tests followed by a correction with Dunn test for multiple comparison with vehicle and # *p* < 0.05 for comparison between conditions with heat-inactivated or active p75NTR antibodies. **g** Representative traces of paired pulse facilitation recordings from slices treated with vehicle (*n* = 6), with heat-inactivated p75NTR antibodies (1/500, *n* = 6) or with active p75NTR antibodies (1/500, *n* = 6). **h** Paired pulse ratios, * *p* < 0.05 One way ANOVA followed by Bonferroni test

We then investigated if proBDNF regulates glutamate release in the EC. We found that a 10 min proBDNF treatment of cortical slices increased the inter-event intervals more than 3-fold (Before proBDNF: 1365.58 +/− 85.58 ms; after proBDNF: 4507.08 +/− 720.91 ms), while only slightly decreasing sEPSC amplitude (Before proBDNF: 22.54 +/− 0.20 pA; after proBDNF: 20.31 +/− 0.33 pA) (Fig. 6a–c). Once elicited by proBDNF, these effects were stable and long-lasting as perfusion with normal ACSF for 10–35 min after proBDNF treatment only partially reduced the inter-event intervals (data not shown).

**Fig. 7:**
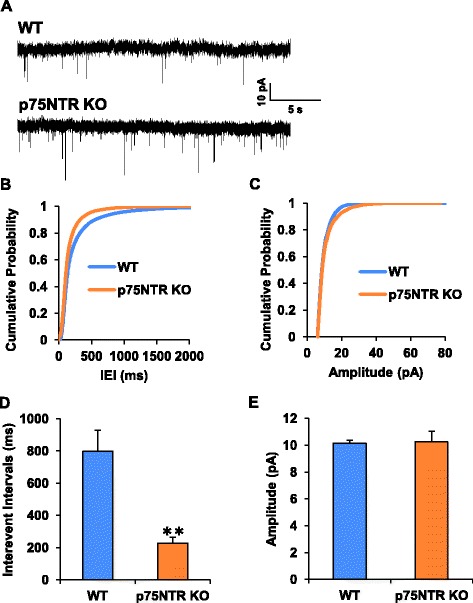
Increased synaptic activity in the entorhinal cortex of p75NTR null mice. **a** Representative traces of mEPSC recordings from cortical slices of wild-type (*n* = 16) or p75NTR null mice (*n* = 11). **b**, **c**. Cumulative plots of interevent intervals and mEPSC amplitudes for both genotypes. **d**, **e**. Mean interevent intervals and current amplitudes in both genotypes. ** *p* = 0.0013, t-test

Since deletion or blockade of p75NTR facilitates persistent firing [27], we hypothesize that this facilitatory effect could reflect a combination of postsynaptic and presynaptic effects. To address this, we measured sEPSCs in slices treated with p75NTR function-blocking antibodies and found that inter-event intervals were reduced by more than 50 %. This effect was attenuated if the antibodies are heat-denatured while amplitude of the sEPSCs remained unchanged (Fig. 6d–f). We also asked if p75NTR blockade had an effect on paired-pulse ratios, an alternate index of neurotransmitter release probability. Treatment with p75NTR-blocking antibodies decreased the paired-pulse ratio by almost 40 % (Vehicle: 1.80 +/−0.16; Heat-inactivated p75NTR Ab: 1.93 +/−0.1; p75NTR Ab: 1.15+/−0.11) whereas heat-inactivated antibodies had no effect in this assay (Fig. 6g–h). To further characterize the role of p75NTR in the regulation of neurotransmitter release, we recorded miniature excitatory post-synaptic currents (mEPSCs) in p75NTR knock-out mice (Fig. 7a). Quantification of the mEPSC shows that deletion of p75NTR shortens the interevent intervals (WT: 799.22 +/−128.30 ms; p75NTR^-/-^: 226.7 +/−36.03 ms, *p* = 0.0013) (Fig. 7b, d) without affecting the amplitude of the mEPSCs (WT: 10.13 +/−0.23 pA; p75NTR^-/-^: 10.25 +/−0.77 pA, *p* = 0.87) (Fig. 7c–e). Altogether, these data strongly suggest that presynaptic p75NTR acts tonically to down regulate glutamate release.

**Fig. 8:**
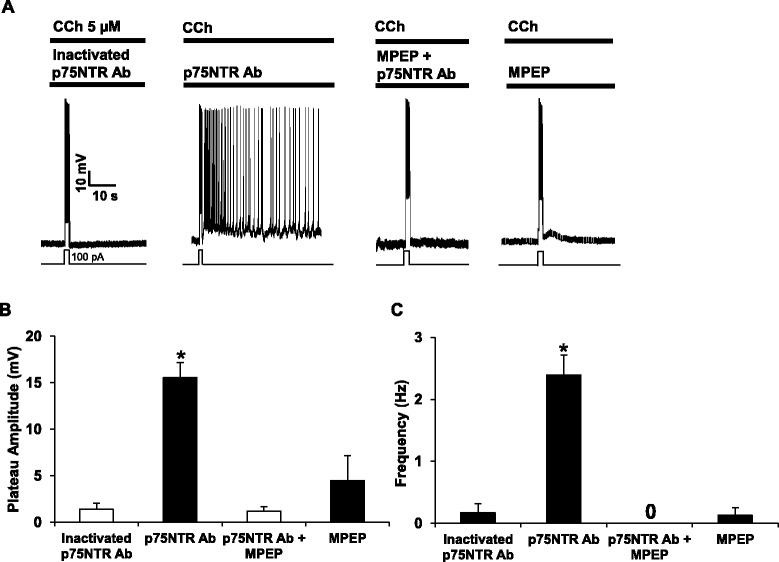
mGluR5 mediates p75NTR-dependent increase in persistent activity. **a** Typical current-clamp recordings of pyramidal neurons of the entorhinal cortex in brain slices treated with heat-inactivated p75NTR antibodies (1/500, 90 min, *n* = 6), with function-blocking p75NTR antibodies (1/500, 90 min, *n* = 5), with MPEP (50 μM, 30 min) followed by an antibody targeting p75NTR (1/500, 90 min) always in presence of MPEP (*n* = 6), or with MPEP alone (50 μM, 2 h, *n* = 6). MPEP effectively suppresses the persistent activity normally observed when p75NTR is blocked. **b**, **c** Quantification of the plateau potential amplitudes and firing frequencies of persistent activity in different conditions. 0 means no firing (0 Hz) in the condition MPEP + p75NTR antibody. * *p* < 0.05, One way ANOVA followed by Bonferroni test

As mentioned earlier, persistent firing can be driven by activation of postsynaptic mGluR5. Since mGluR5 is highly expressed in the entorhinal cortex [32] and since we observed an increase in the release of glutamate when p75NTR is blocked, we hypothesized that mGluR5 may play a major role in the expression of persistent activity in the EC. To address this, we blocked p75NTR with function-blocking antibodies in the cortical slices in the absence or presence of the mGluR5 antagonist MPEP (50 μM) (Fig. 8a). We observed that the cholinergic PF elicited by 5 μM CCh is effectively inhibited by pre-exposure to MPEP. Importantly, plateau potential and firing (Fig. 8b, c), are significantly suppressed when mGluR5 is silenced, indicating the key role of glutamatergic inputs and mGluR5 transduction in the expression of PF in the EC.

## Discussion

**Fig. 9:**
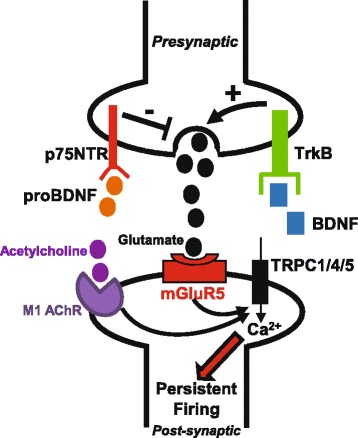
BDNF and proBDNF modulate persistent activity by regulating presynaptic glutamate release in layer V of the entorhinal cortex. BDNF increases glutamate release via presynaptic TrkB activation whereas proBDNF inhibits glutamate release via presynaptic p75NTR receptors. Glutamate acts through postsynaptic Gq-coupled mGluR5 receptors, in addition to acetylcholine acting through M1 muscarinic receptors, to induce calcium entry through TRPC channels and initiation of persistent activity

In the present study, we demonstrated that BDNF facilitates the expression of persistent activity in cortical pyramidal neurons. To our knowledge, it is the first time that BDNF has been shown to regulate this property of sustained activity in the brain. An increased input resistance, a depolarized resting membrane potential and a shifted input/output curve after incubation with BDNF proved that the excitability of principal neurons in layer V of medial EC is upregulated by this neurotrophin. It is interesting to note that the level of expression of the BDNF receptor TrkB in the EC is one of the highest of the hippocampal/parahippocampal region [33]. Based on our results on the excitability state of pyramidal neurons, it would be easy to assume that a post-synaptic mechanism is involved in the potentiating effect of BDNF on cholinergic PF. However, BDNF cannot facilitate PF in presence of the metabotropic glutamate receptor blocker MPEP, indicating that glutamate is a necessary component of this facilitation. Accordingly, BDNF increases the release of glutamate in the EC and this effect is fully inhibited by the TrkB antagonist K252a. We conclude that BDNF acts via presynaptic TrkB to induce the release of glutamate which then acts through mGluR5 to potentiate persistent firing in pyramidal neuron. It has been previously shown that mGluR5 can itself induce persistent firing, using DHPG as a glutamatergic agonist in the anterior cingulate cortex [31]. In our experiments we never found persistent firing induced with BDNF alone and a cholinergic agonist was necessary for the facilitating effect of BDNF. Therefore BDNF is not an inducer of PF per se but acts as regulator of this complex property. BDNF is released in an activity-dependent manner [34] and we propose that activity-dependent BDNF release enhances the release of glutamate in the EC to facilitate the sustained activity of pyramidal neurons (Fig. 9). Interestingly, BDNF is involved in learning and memory processes, including EC-dependent object recognition memory [35], and BDNF levels within the EC increase after acquisition of spatial tasks [18, 36, 37]. Thus, previous studies and this present work show that BDNF facilitates the acquisition of short-term memory.

ProBDNF and BDNF have been proposed to be yin and yang molecules [38, 39] because of their opposing effects in several cellular pathways [40]. We observed that proBDNF controls excitatory synaptic transmission in the EC by inhibiting the release of glutamate. Interestingly, in support of a presynaptic role of proBDNF, the blockade of p75NTR has exactly the opposite effect, increasing the frequency without affecting the amplitude of sEPSCs. Paired-pulse ratio measurements confirmed an increase in release probability-characterized by a large decrease in the paired-pulse facilitation - when p75NTR is blocked. To our knowledge, it is the first time that a role of the p75NTR-proBDNF cascade in the glutamatergic transmission has been demonstrated. Previous reports have touched on this issue in the hippocampus [41], but they did not observe any effect of the global genetic deletion of p75NTR on basal synaptic transmission. However, we have recently shown that in mice with constitutive or conditional p75NTR deletion have deficits in their control of neuronal excitability [27], an effect mimicked pharmacologically using function-blocking p75NTR antibodies. Thus we propose that p75NTR plays a dual regulatory role by controlling excitatory synaptic transmission as well as modulating neuronal excitability, at least in the EC.

The Gq-coupled glutamate receptor mGluR5 is required for facilitation of persistent activity induced by BDNF and modulated by proBDNF. mGluR5 is highly expressed in the medial EC and its role in cognitive processes, including learning and memory has been extensively studied [42]. In layer V pyramidal neurons, mGluR5 is responsible for initiating a long-lasting depolarization underlying persistent firing [43]. The role of mGluR5 in persistent firing was also described in layer 2/3 pyramidal neurons from the EC [44]. Interestingly, PF induced via mGluR5 activation was shown to be regulated post-synaptically by dopamine [43], leading to the hypothesis that mGluR5 blockade with MPEP could be used to treat drug addiction. We reported also that mGluR5-mediated PF can be induced by increasing the release of glutamate with norepinephrine acting on presynaptic α1 adrenoceptors [45]. Our results on BDNF and proBDNF add new insights into the neuromodulation of PF, highlighting the plasticity of this neuronal activity finely tuned by several endogenous factors. Interestingly proBDNF is the major form released in response to physiological stimuli [39, 46]. It is reasonable to speculate that proBDNF plays a key role in regulating information processing by altering the threshold for excitation in neural networks.

## Conclusions

Persistent firing is a property of cortical pyramidal neurons involved in many mnemonic and executive processes. However, the regulation of this activity remains poorly understood. In this study, we show that mature BDNF potentiates persistent firing via its preferred receptor TrkB whereas the precursor form of the neurotrophin, proBDNF, has an opposite effect on synaptic transmission and persistent firing by activating p75NTR. To our knowledge, this is the first time that an endogenous molecule has been shown to facilitate this neuronal activity. This work provides a better understanding of how persistent firing of pyramidal neurons is regulated and provides a molecular framework for deciphering the beneficial effect of BDNF on learning and memory.

## Methods

### Brain slice preparation

All experimental procedures were approved by McGill University Animal Care Committees. Acute brain slices were obtained from 6 to 8 weeks-old male C57BL/6 mice (Charles River Canada, Saint-Constant, Quebec, Canada). Acute mouse brain slices were prepared as previously described [47]. Briefly, mice were anesthetized with ketamine:xylamine cocktail (60 mg/kg) and perfused with ice-cold choline chloride-based artificial cerebrospinal fluid (ACSF) containing (in mM) : 110 choline-Cl, 1.25 NaH_2_PO_4_, 25 NaHCO_3_, 7 MgCl_2_, 0.5 CaCl_2_, 2.5 KCl, 7 glucose, 3 pyruvic acid and 1.3 ascorbic acid, bubbled with carbogen (O_2_ 95 %, CO_2_ 5 %). Semicoronal horizontal slices (300 μM) containing the medial entorhinal cortex (EC) from the retrohippocampal region were obtained using a VT1000 vibratome (Leica, Ontario, Canada) in the same choline chloride-based solution. The slices were allowed to settle down in ACSF containing (in mM) : 124 NaCl, 3 KCl, 26 NaHCO_3,_ 1.8 MgSO_4_, 1.25 NaH_2_PO_4_, 10 glucose,1.6 CaCl_2_ for 1 h before recording at room temperature (22–24 °C).

### Chemicals and solutions

All drugs were purchased from Sigma, except 6-methyl-2-(phenylethynyl) pyridine (MPEP) and K252a from Tocris, mBDNF, proBDNF (wild-type mouse, #B240) and tetrodotoxin (TTX) from Alomone, p75NTR antibody (AB-N01) from Advanced Targeting Systems. All drug stocks were freshly diluted to the desired concentrations. Final concentration of DMSO never exceeded 0.01 %. Extracellular solution in current-clamp and voltage-clamp experiments contained (in mM): 124 NaCl, 3 KCl, 26 NaHCO_3,_ 1.8 MgSO_4_, 1.25 NaH_2_PO_4_, 10 glucose, 1.6 CaCl_2_ and pH was maintained at 7.4 by constant bubbling with carbogen (95 % O_2_, 5 % CO_2_). During persistent firing experiments synaptic transmission was blocked using kynurenic acid (2 mM) and picrotoxin (100 μM).

During mEPSCs recordings, tetrodotoxin (TTX, 1 μM) and picrotoxin (100 μM) were applied and spontaneous activity was recorded during 5 min with the resting membrane potential held at -70 mV. Intracellular solution for current-clamp and voltage-clamp was composed of (in mM): 120 K-gluconate, 10 HEPES, 0.2 EGTA, 20 KCl, 2 MgCl_2_, 7 diTrisP-Creatine, 4 Na_2_ATP and 0.3 NaGTP (pH adjusted to 7.3 with KOH).

### Recording procedures

Brain slices were placed in a recording chamber mounted on the stage of an upright Axioskop microscope (Zeiss, Oberkochen, Germany) equipped with a 63X water immersion objective and differential contrast optics. A near-infrared charged-coupled device (CDD) camera (Sony XC-75) was used to visualize the neurons. Brain slices were stabilized using a U-shaped stainless stell anchor with Lycra threads at 1.5 mm-spacing (warner Instruments LLC, Hamden, CT). Layer V entorhinal neurons selected for recording were located close to *lamina dissecans*. Brain slices were perfused by gravity at a speed of 1–2 ml/min. The temperature of perfusion solution was maintened at 32–34 °C using a TC-324B temperature controller (Warner Instruments, Hamden, CT). Patch pipettes (5-9 MΩ) were pulled on a Brown Flaming puller (P-97, Sutter Instruments, Novato, CA) using borosilicate glass electrode (Sutter Instruments). Tight seals (> 5GΩ) were obtained by applying negative pressure. Electrical signals were amplified using an Axopatch 200B amplifier (Molecular Devices, Sunnyvale, CA), low-pass-filtered at 10 kHz, digitized at 10 kHz via a Digidata 1322A interface (Molecular Devices), and stored on a computer using pClamp 9.2 software (Molecular Devices) for off-line analysis. In this study, all recorded cells displayed a resting membrane potential ranging from −60 to −75 mV. Cells with a resting membrane potential more positive than –55 mV were discarded. In current clamp recordings, the holding current was around 0 pA and slightly adjusted to obtain a membrane potential of –60 mV. Series resistance (<20 MΩ) was not compensated. Input resistance was assessed by injecting negative current pulses (−100 pA, 1 s) at−60 mV. Following whole cell patch, a depolarizing current pulse (100 pA, 1 s) was applied to induce repetitive spiking. Induced persistent firing was defined by its firing frequency and plateau potential amplitude. Firing frequency was measured as the average spiking frequency after the depolarizing current pulse. Plateau potential amplitude (mV) was measured as the difference between the mean membrane potential at baseline within one minute pre-pulse and the mean membrane potential during the sustained phase of the afterdepolarization within 10 s post-pulse. In voltage clamp experiments for recording spontaneous excitatory postsynaptic currents, membrane potential was held at−70 mV (approximate reversal potential of inhibitory postsynaptic current) and series resistance was always compensated (> 70 %). Paired-pulse facilitation (PPF) was measured with ACSF containing 100 μM picrotoxin, stimulation was applied with a bipolar tungsten microelectrode and layer 2/3 of the medial entorhinal cortex was stimulated. PPF was evoked with paired stimuli at intervals of 50 ms. Stimulus intensity was calibrated at 70 % of the maximal response for each cell. PPF was expressed as the ratio of second to first EPSC peak amplitude. Ten traces (trains delivered at intervals of 5 s) from each cell were averaged to measure one PPF value, and only one cell per slice was used.

### Data analysis

Electrophysiological data were analysed using Clampfit 9.2.1.8 (Axon Instruments) and Mini-analysis 6.0.7 (Synaptosoft Inc). mEPSCs were analysed offline using the Mini analysis program (Synaptosoft). Values were expressed as means +/−S.E.M. Statistical analysis was performed with GraphPad prism 5, differences were considered statistically significant when *p* < 0.05 (tests used and p values described in figure legends).

## Abbreviations

Ab: antibody
ACSF: artificial cerebro-spinal fluid
ActD: actinomycin d
BDNF: brain derived neurotrophic factor
CCh: carbachol
CHX: cycloheximide
EC: entorhinal cortex
IEI: inter-event intervals
LTP: long term potentiation
mGluR5: metabotropic glutamate receptor 5
MPEP: 2-methyl-6-(phenylethynyl)pyridine hydrochloride
NGF: nerve growth factor
NT3: neurotrophin 3
NT4/5: neurotrophin 4/5
p75NTR: p75 neurotrophin receptor
PF: persistent firing
PPF: paired-pulse facilitation
proBDNF: pro-brain derived neurotrophic factor
sEPSC: spontaneous excitatory post-synaptic current
TrkB: tyrosine receptor kinase b
TRP: transient receptor potential
TTX: tetrodotoxin
VPS10: vacuolar protein sorting 10
